# Quality of total hip arthroplasty health care based on four years of patient-reported outcomes in the Netherlands

**DOI:** 10.1186/s12955-023-02104-2

**Published:** 2023-03-14

**Authors:** Yvette Pronk, Walter van der Weegen, Berend Willem Schreurs, Peter Pilot

**Affiliations:** 1 Research Department, Kliniek ViaSana, Hoogveldseweg 1, 5451 AA Mill, The Netherlands; 2 Department of Orthopaedic Surgery, Sports & Orthopaedics Research Centre, Sint Anna Ziekenhuis, Bogardeind 2, 5664 EH Geldrop, The Netherlands; 3 Department of Orthopaedic Surgery, Radboudumc, Geert Grooteplein Zuid 10, 6562 GA Nijmegen, The Netherlands; 4 Landelijke Registratie Orthopedische Implantaten (LROI), Bruistensingel 230, 5232 AD ’s-Hertogenbosch, The Netherlands; 5 IMUKA, Kanaalstraat 10, 6116 AD Roosteren, The Netherlands

**Keywords:** Patient reported outcome measures, PROMs, Total hip arthroplasty, Quality of health care, Improvement

## Abstract

**Background:**

Joint arthroplasty registries have incorporated patient-reported outcomes (PROs) to evaluate outcomes from a patients’ perspective to improve total hip arthroplasty (THA). To draw valid conclusions on PROs, a minimum response rate (RR) of 60% is advised. This study investigated (1) if the quality of THA health care based on PROs improved over the years in the Netherlands, (2) if RRs improved over the years, and (3) difference in PROs over the years in hospitals with RR ≥ 60% compared to RR < 60%.

**Methods:**

Longitudinal study with publicly available datasets from 2016 to 2019. Primary outcome was increase/decrease in PRO change scores including 95%CI ranges over the years between preoperatively and 3 months postoperatively (pre-3 m), and 12 months postoperatively (pre-12 m). Improved quality of health care was arbitrary defined as when ≥ 3 of 4 included scores or ranges were statistically significant improved. Secondary outcome was increase/decrease in RRs over the years. Subgroups RR ≥ 60% and RR < 60% were compared.

**Results:**

Hospitals (%) collecting THA PROs increased from 78 to 92%. EQ VAS change score increased over the years, and 95%CI ranges of EQ VAS, EQ-5D descriptive system and NRS pain during activity decreased over the years at pre-3 m (*p* < 0.05). All THA pre-12 m PRO change scores and 95%CI ranges remained equal (*p* > 0.05). Pre-3 m RR remained equal (around 43%, *p* = 0.107) and pre-12 m RR decreased 9% (49% to 40%, *p* = 0.008). Pre-3 m subgroup RR ≥ 60% was too small to analyse (5%). No difference was found between pre-12 m subgroups (RR ≥ 60% = 16%), *p* > 0.05).

**Conclusions:**

Quality of THA health care based on PROs seems equal in the Netherlands between 2016 and 2019. Although more hospitals participated in PRO collection, low RRs with large IQRs are observed and only 16% of the hospitals achieved the advised RR ≥ 60%. Multiple recommendations are provided to improve PRO collection and use.

## Background

Total hip arthroplasty (THA) is an effective treatment for patients with end-stage hip osteoarthritis. Traditionally, THA is surgically successful if alignment is correct, and the implant well fixed and stable. The long term outcome is considered optimal if excellent implant survival is obtained. However, patients are mainly satisfied if their pain is relieved, their function is restored, their quality of life has improved and they can participate in daily activities. To measure these outcomes, collection of patient-reported outcomes (PROs) by selected patient-reported outcome measures (PROMs) has become an internationally accepted method.

Multiple national joint arthroplasty registries have incorporated PROs to evaluate the outcomes from a patients’ perspective to improve THA health care [1–3]. The Dutch arthroplasty register (LROI) incorporated PROs of patients diagnosed with hip osteoarthritis since 2014. In the Netherlands, these PROs are also a mandatory part of a national defined indicator set since 2016. These results are publicly available to create transparency of the delivered care [4]. To improve health care, hospitals could use these publicly available PROs to benchmark themselves. Furthermore, surgeons could use these data to inform their patients what to expect of a treatment and to facilitate shared decision making. Moreover, health insurance companies could use PROs in their negotiations with hospitals. However, previous studies emphasize that there is no definitive evidence yet that the goal of improving health care by evaluating PROs is achieved [5–9].

Informing patients on what PRO results to expect, discussing with patients what PRO results are achieved and pro actively following up on deviating PRO results are examples of how to incorporate PROs in daily practice, which might lead to improved quality of THA health care from the patients’ perspective.

Collecting PROs to adequately evaluate THAs involves effort and budget [10]. Nowadays, 50% of the world-wide existing national joint arthroplasty registries capture preoperative and postoperative PROs of the patients [3]. Multiple national joint arthroplasty registries do not achieve the advised minimum RR of 60% yet [3, 11, 12]. So, investing effort and budget to collect these data in its current form could be questioned, especially if it is unclear if the quality of health care is improved by collecting and using PROs.

It was hypothesized that evaluating PROs will result in improved quality of THA health care from a patients’ perspective, which should be reflected in better PROs and higher RRs over the years. Therefore, the primary aim of this study was to investigate if the quality of THA health care from a patients’ perspective based on PROs improved over the years since the mandatory introduction of the PROM indicators in the Netherlands in 2016. Secondary aims were to investigate (1) if PROM RRs improved over the years, and (2) if there was a difference in PROs over the years between hospitals which achieved the advised minimum RR of 60% compared to hospitals that did not. Better PROs from hospitals with a RR ≥ 60% were expected.

## Methods

For this longitudinal study, the publicly available Dutch national THA indicator datasets were downloaded (https://www.zorginzicht.nl/openbare-data/open-data-medisch-specialistische-revalidatie). Datasets were included from the start of the PROM indicators in 2016 up to and including 2019. Although the datasets of 2020 and 2021 were available, these datasets were not included due to unknown effect of the COVID-19 pandemic on the quality of health care.

In case of hospitals with multiple locations, these locations were considered as separate entities. Hospitals were included when they were present in all included datasets. Reasons for not being present in all included datasets could be merging of hospitals, bankruptcy or newly hospitals started up after 2016. Hospitals were excluded when in the data quality rapports, published by a governmental institution (Zorginstituut Nederland, Diemen, the Netherlands) each year [13], problems with the data quality was mentioned, for example: two locations of one hospital sent in the same scores.

### Dutch national indicator datasets

The PROM indicators are part of the Dutch national THA indicator dataset. The PROM indicators are (1) the preoperative response rate, (2) the preoperative score per PROM and (3) the change scores between preoperative and multiple postoperative measurement time points per PROM [14]. The THA PROM set used is the mandatory PROM set of the Dutch Orthopaedic Association [15]. Hospitals had to collect or upload the PROs for all patients diagnosed with hip osteoarthritis in the Dutch arthroplasty register (LROI). The Dutch arthroplasty register data scientists calculated the numbers of the PROM indicators including correction for case mix (gender, age, Charnley score, smoking, ASA, preoperative PRO and BMI) when calculating change scores. This method was the same for all hospitals. Hospitals were asked to verify the data, which, after approval, were sent to Zorginstituut Nederland. This institution published the datasets online.

From these datasets the following data were collected per year, per hospital, per preoperative or change PROM measurement time point and per PRO: number of THAs with a score, mean score, 95% confidence interval (95%CI) lower bound and 95%CI upper bound. Furthermore, per year and per hospital the number of performed primary THAs, and the number of surgeons performing these surgeries were collected. The numbers of performed THA and surgeons were based on all THA patients, not only on patients diagnosed with hip osteoarthritis (85% of all THA patients) [16].

### Outcomes

The primary outcome was the increase or decrease in PRO change scores including 95%CI ranges over the years. The four included PROs were pain at rest, pain during activity, quality of life and physical functioning. Pain at rest and pain during activity were both measured using a Numeric Rating Scale (NRS) question scored from 0 (no pain) to 10 (severe pain). NRS are well correlated and sensitive for pain assessment including osteoarthritic knee pain and are preferred over Visual Analogue Scales by the elderly population [17–19]. A decrease in the score was defined as an improvement in these PROs over the years. Quality of life was assessed with 3-level version of EuroQol 5 dimensions questionnaire (EQ-5D-3L) which existed of two subscores: EQ-5D descriptive system with the highest score 1 defined as healthy, and EQ visual analogue scale (EQ VAS) scored from 0 (worst imaginable health state) to 100 (best imaginable health state) [20]. An increase in both subscores was defined as an improvement in this PRO over the years. Physical functioning was measured using Hip disability and Osteoarthritis Outcome Score-Physical Function Shortform (HOOS-PS) on a scale from 0 (no difficulty) to 100 (extreme difficulty) [21, 22]. Although HOOS-PS has to be used with care, it was a mandatory PRO from the 2012 guideline on PRO collection from the Dutch Orthopedic Association [1, 23]. A decrease in this score was defined as an improvement in this PRO over the years. The 95%CI range was calculated by 95%CI upper bound minus 95%CI lower bound. A decreased 95%CI range was defined as an improvement over the years. The included change scores and 95%CI ranges were between preoperatively and 3 months postoperatively (pre-3 m), and between preoperatively and 12 months postoperatively (pre-12 m). As a minimal clinically important difference (MCID) is not available for most PROs [24, 25] and to answer the primary aim based on the same method per PRO, improved quality of health care over the years was defined as when ≥ 3 of the 4 included PRO change scores or 95%CI ranges were statistically significant improved over the years. As the EQ-5D descriptive system and EQ VAS were two subscores of one PROM for one PRO, both counted for 0.5.

The first secondary outcome was the increase or decrease in PROM RRs over the years. RR was calculated by dividing the highest number of performed THAs with a PRO preoperative score or change score by the number of performed THAs multiplied by 0.85 and, thereafter, multiplied by 100. By multiplying with 0.85 a correction was made for the difference between the number of performed THAs (all patients) and the number of performed THAs with a PRO score (patients diagnosed with hip osteoarthritis, 85% [16]). RR was calculated for response on the preoperative measurement (pre RR), for response on both preoperatively and 3 months postoperatively measurements (pre-3 m RR), and for response on both preoperatively and 12 months postoperatively measurements (pre-12 m RR). The second secondary outcome was increase or decrease in PRO change scores including 95%CI ranges over the years between hospitals which achieved the advised minimum RR of 60% and hospitals that did not. Per calculated RR, hospitals were allocated to subgroup RR ≥ 60% or subgroup RR < 60%. Hospitals needed to have a RR ≥ 60% in all four years for allocation to the subgroup RR ≥ 60%.

### Statistical analysis

Based on the data quality rapports published by Zorginstituut Nederland, unlikely outliers were recoded into missing values. Statistical analyses were performed using SPSS version 26.0 (IBM Corp, Armonk, New York). Results were reported in mean and standard deviation (SD), median and interquartile range (IQR) or number (n) and percentage (%) based on the test performed.

Differences in the number of performed THAs and the number of surgeons performing these surgeries between included and excluded hospitals were investigated. Distribution of the data was investigated using Shapiro–Wilk tests of normality. Mann–Whitney U tests were used for these non-parametric distributed data.

Of the included hospitals, for each PRO at pre-3 m or pre-12 m, normal distribution of the change score and 95%CI range were investigated using Shapiro–Wilk tests of normality. For the primary aim change score and 95%CI range of each PRO at pre-3 m or pre-12 m were analysed on the overall rate of increase or decrease over the years using linear mixed model analyses. For the secondary aims linear mixed model analyses were executed to investigate the overall rate of increase or decrease of PROM RR over the years for each RR, and to investigate the overall rate of increase or decrease of each PRO change score and 95%CI range between both subgroups. When the percentage of included hospitals in the subgroups RR ≥ 60% or RR < 60% were below 10%, these analyses were not executed. The linear mixed model analyses included correction for differences between included and excluded hospitals. Continuous variables were centralized to create a more interpretable intercept.

## Results

**Fig. 1:**
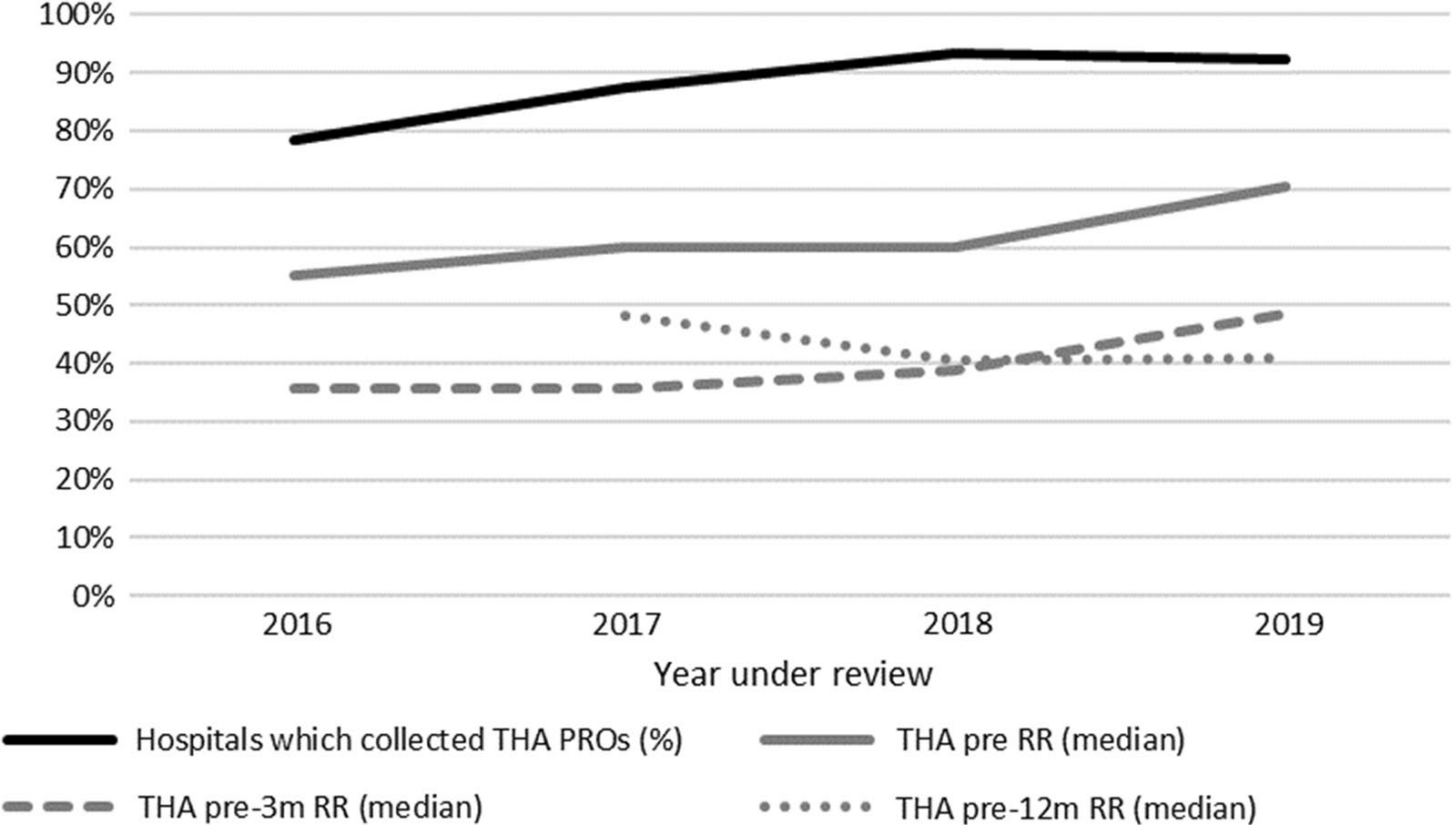
Hospitals which collected THA PROs, and THA PROM RR per measurement time point per year. In 2016, pre-12 m RR data was not available yet. Pre = preoperative; Pre-12 m = between preoperatively and 12 months postoperatively; Pre-3 m = between preoperatively and 3 months postoperatively; PROs = patient-reported outcomes; RR = response rate; THA = total hip arthroplasty

Between 2016 and 2019 124,810 THAs were implanted. In these four years THA data of 109 unique hospitals were published. This number of 109 is partly based on merging hospitals and new hospital registrations. The number of hospitals per year was rather constant: mean 92 hospitals per year (2016: 92, 2017: 95, 2018: 91, 2019: 90). The number of hospitals collecting PROs increased from 72 (72/92, 78%) in 2016 to 83 (83/90, 92%) in 2019. Median pre RRs were between 55% (IQR 39%) and 70% (IQR 38%), median pre-3 m RRs were between 36% (IQR 32%) and 48% (IQR 33%) and median pre-12 m RRs were between 41% (IQR 43%) and 48% (IQR 55%) (Fig. 1).

### Included hospitals

Out of mean 92 hospitals per year, 73 (79%) hospitals were included for further analyses. Main reason for exclusion was that no data was available in one or more years (21%). Most of these hospitals (12%) missed more than one year of data. Included hospitals performed statistically significant more THAs by statistically significant more surgeons compared to excluded hospitals (THAs: 352 (240–503) versus 147 (36–238), *p* < 0.001; surgeons: 5 (4–7) versus 3 (2–5), *p* < 0.001).

#### Main results

Of the 4 THA PRO change scores and 95%CI ranges at pre-3 m, EQ VAS change score increased over the years (0.5 of 4) (*p* = 0.008) defined as EQ VAS change score improved over the years. The 95%CI ranges of EQ-5D-3L (both EQ VAS and EQ-5D descriptive system) and NRS pain during activity decreased over the years (2 of 4) (all *p* < 0.001) defined as these 95%CI ranges improved over the years. All THA PRO change scores and 95%CI ranges remained equal over the years at pre-12 m (*p* > 0.05) (Table 1).

**Table 1:** Median change scores including median 95%CI ranges per PRO and per year

	Pre-3 m					Pre-12 m				
	2016	2017	2018	2019	*p* value*	2016	2017	2018	2019	*p* value*
Hospitals (n (%))	55 (75.34)	68 (93.15)	68 (93.15)	67 (91.78)		n.a	64 (87.67)	68 (93.15)	69 (94.52)	
NRS pain at rest (change score (95%CI range))	4.06 (0.87)^a^	4.08 (0.93)	4.02 (0.84)	4.05 (0.70)^e^	0.545 (0.101)	n.a	4.28 (0.85)^b^	4.28 (0.84)^f^	4.33 (0.74)^g^	0.446 (0.053)
NRS pain during activity (change score (95%CI range))	5.08 (1.05)^a^	5.09 (0.72)	5.10 (0.67)	5.07 (0.53)^e^	0.377 (< 0.001)	n.a	5.76 (0.62)^b^	5.81 (0.62)^f^	5.76 (0.59)^g^	0.623 (0.081)
HOOS-PS (change score (95%CI range))	29.38 (7.00)^a^	30.76 (6.30)	30.60 (6.45)	30.19 (5.43)	0.066 (0.078)	n.a	34.72 (5.79)^d^	34.57 (5.52)	35.26 (5.44)	0.694 (0.300)
EQ-5D descriptive system (change score (95%CI range))	0.250 (0.130)^a^	0.250 (0.095)	0.260 (0.090)^f^	0.250 (0.070)	0.090 (< 0.001)	n.a	0.290 (0.080)^d^	0.290 (0.080)^f^	0.280 (0.080)^f^	0.281 (0.220)
EQ VAS (change score (95%CI range))	8.97 (11.90)^a^	10.41 (5.70)^f^	10.97 (5.91)^f^	11.28 (4.59)	0.008 (< 0.001)	n.a	10.57 (5.92)^c^	10.64 (5.40)^f^	11.53 (5.55)^f^	0.603 (0.183)

^*^p values of the overall rate of increase or decrease over the years are presented

EQ-5D descriptive system = EuroQol 5 dimensions descriptive system; EQ-5D VAS = EuroQol visual analogue scale; HOOS-PS = Hip disability and Osteoarthritis Outcome Score-Physical Function Shortform; n.a. = not available as PROs collection was started the year before, so no 12 month scores were available yet; NRS = numeric rating scale; Pre-12 m = between preoperatively and 12 months postoperatively; Pre-3 m = between preoperatively and 3 months postoperatively

^a^n = 54, ^b^n = 61, ^c^n = 62, ^d^n = 63, ^e^n = 66, ^f^n = 67, ^g^n = 68

**Fig. 2:**
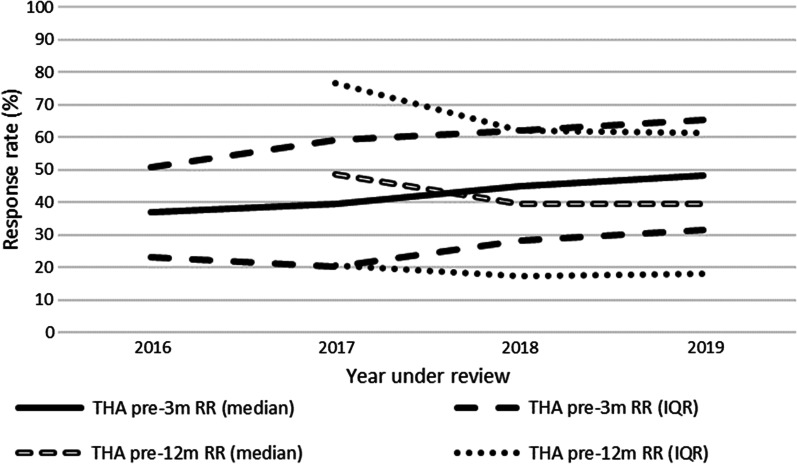
THA PROM RR per measurement time point per year of included hospitals. In 2016, pre-12 m RR data was not available yet. Pre-12 m = between preoperatively and 12 months postoperatively; Pre-3 m = between preoperatively and 3 months postoperatively; RR = response rate; THA = total hip arthroplasty

The pre-3 m RR remained equal (*p* = 0.107) and pre-12 m RR decreased over the years (*p* = 0.008) (Fig. 2). At pre-3 m the subgroup RR ≥ 60% was too small (n = 4, 5%) to answer the second secondary study aim. At pre-12 m the subgroup RR ≥ 60% (16%) reported equal PRO change scores and 95%CI ranges over the years compared to the subgroup RR < 60% (*p* > 0.05) (Table 2).

**Table 2:** Median change scores including median 95%CI ranges in subgroups RR ≥ 60% and RR < 60% at pre-12 m

	RR ≥ 60% (n = 12 (16%))			RR < 60% (n = 57 (78%))			*p* value*
	2017	2018	2019	2017 (n = 52)	2018 (n = 56)	2019 (n = 57)	
NRS pain at rest (change score (95%CI range))	4.31 (0.55)	4.35 (0.52)	4.27 (0.53)	4.26 (0.94)	4.27 (1.00)	4.34 (0.82)	0.673 (0.610)
NRS pain during activity (change score (95%CI range))	5.80 (0.43)	5.74 (0.43)	5.73 (0.43)	5.75 (0.73)	5.83 (0.76)	5.76 (0.67)	0.885 (0.678)
HOOS-PS (change score (95%CI range))	34.08 (3.70)	35.67 (3.54)	35.79 (3.60)	34.72 (7.35)	34.48 (6.46)	35.26 (5.87)	0.378 (0.339)
EQ-5D descriptive system (change score (95%CI range))	0.290 (0.050)	0.290 (0.050)	0.285 (0.055)	0.290 (0.100)	0.290 (0.100)	0.280 (0.090)	0.758 (0.218)
EQ VAS (change score (95%CI range))	9.12 (3.91)	11.82 (3.86)	11.68 (3.79)	10.69 (6.46)	10.51 (6.25)	11.53 (5.81)	0.522 (0.079)

^*^p values of the difference in the subgroups on overall rate of increase or decrease over the years are presented

EQ-5D descriptive system = EuroQol 5 dimensions descriptive system; EQ-5D VAS = EuroQol visual analogue scale; HOOS-PS = Hip disability and Osteoarthritis Outcome Score-Physical Function Shortform; NRS = numeric rating scale; RR = response rate

#### Detailed results: PRO change score and 95%CI range

At pre-3 m EQ VAS change score increased statistically significant over the years (intercept: 10.67 (9.47–11.87), 2016: − 2.25 (− 3.69 to − 0.81), 2017: − 1.52 (− 2.91 to − 0.14, 2018: 0.09 (− 1.03–1.21), 2019: 0; *p* = 0.008). Furthermore, EQ VAS 95%CI range significantly decreased over the years (intercept: 6.44 (5.48–7.41), 2016: 10.61 (6.50–14.72), 2017: 1.98 (0.61–3.36), 2018: 0.54 (− 0.18–1.26), 2019: 0; *p* < 0.001). EQ-5D descriptive system 95%CI range significantly decreased over the years (intercept: 0.107 (0.087–0.127), 2016: 0.080 (0.036–0.123), 2017: 0.015 (− 0.016–0.045), 2018: 0.015 (− 0.006–0.035), 2019: 0; *p* < 0.001). For NRS pain during activity, the 95%CI range significantly decreased over the years (intercept: 0.74 (0.64–0.83), 2016: 0.82 (0.41–1.24), 2017: 0.30 (0.05–0.55), 2018: 0.11 (0.06–0.17), 2019: 0; *p* < 0.001). All PRO change scores and 95%CI ranges remained equal over the years at pre-12 m (Table 1).

#### Detailed results: PROM response rate

The number of hospitals collecting THA PROs increased from 55 (75%) in 2016 to 67 (92%) in 2019. The pre-3 m RR remained equal over the years (around 43%, *p* = 0.107). The pre-12 m RR statistically significant decreased over the years from 49% (IQR 56%) in 2017 to 40% (IQR 43%) in 2019 (intercept: 43.96 (37.65–50.27), 2017: 8.00 (0.87–15.13), 2018: 0.08 (− 4.82–4.98), 2019: 0; *p* = 0.008) (Fig. 2).

#### Detailed results: subgroup response rate ≥ 60% compared to subgroup response rate < 60%

The subgroup RR ≥ 60% comprised of a minimum of 8 (11%) to a maximum of 22 (30%) hospitals per year at pre-3 m, and a minimum of 22 (30%) to a maximum of 27 (37%) hospitals per year at pre-12 m. In total 4 (5%) hospitals reached RR ≥ 60% all years at pre-3 m and 12 (16%) hospitals all years at pre-12 m. At pre-3 m the subgroup RR ≥ 60% was too small to answer the second secondary study aim. At pre-12 m all PRO change scores and 95%CI ranges remained equal over the years between both subgroups (*p* > 0.05). In each year median PRO 95%CI ranges were smaller in the subgroup RR ≥ 60% compared to the subgroup RR < 60% (Table 2).

## Discussion

The primary aim of this study was to investigate if the quality of THA health care from a patients’ perspective based on PROs improved over the years since the mandatory introduction of the PROM indicators in the Netherlands in 2016. Secondary aims were to investigate (1) if PROM RRs improved over the years, and (2) if there was a difference in PROs over the years between hospitals which achieved the advised minimum RR of 60% compared to hospitals that did not. Main results show that of the 4 THA PRO change scores, only EQ VAS change score improved over the years (0.5 of 4) at pre-3 m. Regarding their 95%CI ranges, EQ VAS, EQ-5D descriptive system and NRS pain during activity improved over the years (2 of 4). At pre-12 m all THA PRO change scores and 95%CI ranges remained equal over the years. These results mean that since the mandatory introduction of the PROMs the quality of THA health care from a patients’ perspective based on PROs remained equal at both pre-3 m and pre-12 m (< 3 of 4). Although the percentage of hospitals collecting PROs increased, low RRs with large IQRs were observed. The pre-3 m RR remained equal and, disappointingly, the pre-12 m RR decreased over the years. At pre-3 m the subgroup with sufficient PROs at all years (RR ≥ 60%) was very small (5%) hampering the second secondary aim. Interestingly, at pre-12 m this subgroup (16%) reported equal PRO change scores and 95%CI ranges over the years compared to the subgroup without sufficient PROs (RR < 60%).

The quality of THA health care from a patients’ perspective based on PROs remained equal over the years in the Netherlands between 2016 and 2019, while improvement of quality of health care is the desirable direction. Maybe more years are needed to achieve a detectable improvement. However, a previous single center cohort study on twenty year data of Dutch THA patients executed trends over time analyses and also reported, in general, no improvement over time [26]. Interestingly, in the present study, two PRO 95%CI ranges (EQ-5D-3L (both EQ VAS and EQ-5D descriptive system) and NRS pain during activity) decreased over the years at pre-3 m. Decreased 95%CI ranges mean smaller 95%CI ranges, so less positive and negative outliers, which could be interpreted as an improvement. However, decreased 95%CI ranges could also be the result of more hospitals collecting PROs as more data generally results in smaller 95%CI ranges [27].

The statistical power of large datasets, as is common in data retrieved from national joint registries, has inherent pitfalls. This includes the possibility of reaching statistical significance for a score difference, with this score difference being (much) smaller than the minimal clinical relevant difference, which is the only relevant outcome from the perspective of the patient.

It was hypothesized that PRO collection and transparency of PROs lead in PRO evaluation, which will result in improved future PROs and subsequently improved health care. However, it remains unknown if hospitals use the collected PROs to evaluate (and improve) health care. Collection is mandatory, however, using aggregated or individual PROs in daily practice to evaluate THA health care is not. For evaluation an intrinsic motivation of surgeons, hospitals and other stakeholders is needed [28]. The Dutch Orthopaedic Association uses implant information from the Dutch arthroplasty register (LROI) for an outlier analysis including conversations with hospitals if needed [29, 30]. It is recommended to include an outlier procedure on PROs and RRs. If hospitals only collect to comply with mandatory PRO collection, no better understanding of the patients’ perspective nor improvement of quality of health care will be likely, while the costs and burden involved with PRO collection remain.

With and without excluded hospitals, low median pre-3 m RRs and pre-12 m RRs (< 49%) were observed which indicates low quality of PRO data. Improvement is seen in the percentage of hospitals collecting PROs (around 15%). However, of the included hospitals, pre-12 m RR decreased 9% over the years which is worrisome. Besides the low RRs, large IQRs (56%) were observed. This reveals a large diversity in PRO collection in the Netherlands. To comply with mandatory PRO collection for registries and the Dutch PROM indicators, hospitals need a minimum RR of only 1%. However, there is evidence that for a sufficient evaluation of THAs a minimum RR of 60% is advised [11, 12]. A first exploration by the present study shows that hospitals achieving this 60% at pre-12 m have equal PRO change scores and 95%CI ranges over the years compared to hospitals that do not. Interestingly, PRO 95%CI ranges seem twice as small for hospitals with a RR ≥ 60%. This indicates that less outliers are expected in hospitals achieving RR ≥ 60%. However, these results are based on aggregated scores per hospital per year. Further analyses on individual scores per patient per hospital per year are needed before conclusions on differences between hospitals achieving RR ≥ 60% and RR < 60% could be made.

The low quality of PRO data based on RR is a point of concern. Only 5% of the hospitals achieved the advised RR ≥ 60% at pre-3 m and only 16% at pre-12 m. Therefore, it is questionable if a conclusion on quality of THA health care from a patients’ perspective based on PROs over the years could be made. Continuing PRO collection in its current form, including the involved effort and costs, might not be justifiable from an ethical and value-based health care perspective.

So, in what direction should PRO collection and use develop to improve quality of THA health care from a patients’ perspective? Firstly, investigate if stakeholders use collected PROs to evaluate THA health care. It is assumed that if PROs are made available, they will be used. However, studies examining this assumption have found limited use of PROs. Main reasons according to surgeons are a lack of knowledge on how to use PROs in daily health care, the perception that PROs do not provide actionable information, and because gathering and handling of PROs add work to an already busy schedule [31, 32]. In addition, orthopaedic surgeons state that using PROs on an individual patient level is difficult based on logistical barriers (access and display issues, time required) and perceptual barriers (concerns about patients understanding, and validity and reliability of measures). They prefer to talk with patients about personal outcomes. However, they mention that using PROs on an aggregated level is valuable for hospitals and individual surgeons [33]. Secondly, support stakeholders to evaluate THA outcomes from a patients’ perspective using the already existing multiple examples and recommendations how to use the PROs [34, 35]. Thirdly, investigate how all stakeholders rate the quality of THA health care provided today. Of course, improvement is always desirable, however, there might be a consensus that the delivered quality is of such a high level that improvement is unlikely or that the desired improvement is not value-based. Fourthly, increase the RRs to at least 60% to improve the data quality. Multiple recommendations to improve RRs already exist [10, 36–42]. Fifthly, evaluate the set aim(s) of PROs. Maybe the goal of improving health care from a patients’ perspective is not achievable or not formulated well. Each aim sets different requirements for the PRO(M)s, time points of collecting PROs and statistical analysis. The primary aim is the basis. Although PROMs are the gold standard to measure outcomes from a patients’ perspective at this moment, maybe other instruments are needed to achieve the goal set. These five points need to be part of a coordinated effort of all stakeholders to improve PRO collection and use.

As a strength of the present study, a first exploration is presented on the goal of improving THA health care by evaluating outcomes from a patients’ perspective in the Netherlands. Moreover, as the Dutch arthroplasty registry reported comparable results to multiple other national joint arthroplasty registries [3], similar results are expected for PRO collection in other countries around the world. In a previous review of registry-based studies reporting PRO response rates there was also concern on the large variation and downward trend of PROM response rates [43].

Furthermore, each year the same method for the calculated data in the used public available datasets was used including correction for case mix. As a limitation, due to these used public available datasets, data on if hospitals use the collected PROs to evaluate and, if necessary, to improve their health care were missing. Moreover, only aggregated data of hospitals were available. Furthermore, as a MCID is not available for most PROs [24, 25], the authors needed to define improved quality of health care over the years from a statistical perspective. Future studies should focus on if stakeholders use collected PROs to evaluate THA health care, how all stakeholders rate the quality of health care provided today and if other instruments instead of PROMs are needed to achieve the goal of improving health care from a patients’ perspective.

## Conclusions

The quality of THA health care from a patients’ perspective based on PROs seems equal in the Netherlands between 2016 and 2019. Although the percentage of hospitals collecting THA PROs increased, low RRs with large IQRs reveal a large diversity in PRO collection. Only 16% of the Dutch hospitals have sufficient PROs to evaluate THAs from a patients’ perspective at 12 months (RR ≥ 60%). Based on these observations, it is questionable if a conclusion on quality of THA health care based on PROs could be made. Similar results are expected for PRO collection in other countries around the world. Multiple recommendations are provided to improve PRO collection and use. A coordinated effort of all stakeholders should be initiated to improve PRO collection and use.

## Data Availability

The datasets used and analysed during the current study are publicly available (Zorginstituut Nederland; https://www.zorginzicht.nl/openbare-data/open-data-medisch-specialistische-revalidatie).

## Abbreviations

pre-12 m: Between preoperatively and 12 months postoperatively
pre-3 m: Between preoperatively and 3 months postoperatively
EQ VAS: EQ visual analogue scale
EQ-5D-3L: EuroQol 5 dimensions questionnaire
HOOS-PS: Hip disability and Osteoarthritis Outcome Score-Physical Function Shortform
MCID: Minimal clinically important difference
NRS: Numeric rating scale
PROMs: Patient-reported outcome measures
PROs: Patient-reported outcomes
RR: Response rate
THA: Total hip arthroplasty
